# Agmatine Abrogates Tacrolimus-Induced Testicular Injury in Rats

**DOI:** 10.3390/pharmaceutics17060703

**Published:** 2025-05-27

**Authors:** Naif Alharbi, Omnia Nour, Mirhan N. Makled, Manar Nader

**Affiliations:** Department of Pharmacology and Toxicology, Faculty of Pharmacy, Mansoura University, Mansoura 35516, Egypt; naif_alharbi7@hotmail.com (N.A.); mirhan_makled@mans.edu.eg (M.N.M.); manarahna@yahoo.com (M.N.)

**Keywords:** agmatine, oxidative stress, tacrolimus, testicular toxicity

## Abstract

**Background/Objectives:** Tacrolimus is an immunosuppressant drug widely used to prevent organ transplant rejection. Preclinical and clinical studies report that tacrolimus has destructive impacts on the male reproductive system owing to the induction of oxidative stress and inflammation. This study aimed at examining defensive impacts of agmatine against tacrolimus-induced testicular toxicity in rats. **Methods:** Male Wistar rats were randomly divided into six groups and treated based on the experimental design for 14 days. By the end of this study, blood samples were obtained to measure testosterone and luteinizing hormone. Also, both testes were removed for molecular analysis and histopathological examinations. **Results**: Agmatine administration increased serum levels of testosterone and luteinizing hormone and ameliorated all histopathological and toxicological changes induced by tacrolimus. Agmatine administration attenuated tacrolimus-induced oxidative stress as evidenced by the reduction of malondialdehyde content and inducible nitric oxide synthase expression and the elevation of reduced glutathione. This was parallel to the restoration of nuclear factor erythroid 2-related factor2 and hemeoxygenase-1 expression. Moreover, agmatine decreased the expressions of nuclear factor kappa B and interleukin-17. Agmatine also decreased the cell death revealed by decreased caspase-3 expression and increased expression of the antiapoptotic marker Bcl-2 in a dose-dependent manner. The antioxidant, anti-inflammatory, and antiapoptotic effects of agmatine were explained by increased expression of sirtuin-1. **Conclusions**: agmatine effectively attenuated testicular injuries induced by tacrolimus and enhanced spermatogenesis. This protective effect of agmatine might be mediated via the upregulation of sirtuin-1 expression that in turn restores oxidative status and regulates nuclear factor erythroid 2-related factor2/nuclear factor kappa B/Bcl-2 signaling.

## 1. Introduction

Tacrolimus (TAC), known as FK506, is a potent calcineurin inhibitor that is widely used to prevent organ rejection post-transplant and to treat severe atopic dermatitis [1,2]. The majority of studies have focused only on preventing toxic effects of TAC on the liver, kidney, and neurons, as well as the gastrointestinal tract [3,4]. Evidence reports that males on TAC therapy suffering from testicular injuries including a loss of epithelial thickness in seminiferous tubules, an increase in the number of spermatids and spermatozoa with damaged heads and tails, and a decrease in the number of spermatocytes [5,6]. However, very few studies have focused on the toxic effects of TAC on testicular damage and possible therapeutic tools that could help prevent the testicular toxicity associated with TAC therapy.

TAC exerts its immuno-suppressant activity through inhibiting interleukin (IL)-2 mRNA transcription via forming a pentameric complex with the intracellular receptor, FKBP12, Ca^2+,^ calmodulin, and calcineurin [7]. FKBP12, which is a major pharmacological target molecule of TAC, has an important role in sperm motility and could be related to the TAC-induced decrease in sperm motility [8].

Reactive oxygen species (ROS) overproduction and diminished ATP production associated with impaired mitochondrial function have been proposed to be the main causes of TAC-associated organ toxicity [9]. Thus, blocking oxidative stress and maintaining energy homeostasis could be a potential mean of reducing the organ toxicity associated with TAC.

Agmatine (AGM) is an endogenous metabolite of L-arginine [10]. Preclinical and initial clinical evidence has shown that AGM could treat opioid addiction, neurodegenerative diseases, mood disorders, cognitive disorders, and neurotrauma [11,12]. AGM is known to have potent antioxidant and anti-inflammatory effects through inhibiting inducible nitric oxide synthase (iNOS) and activating endothelial nitric oxide synthase (eNOS) [13,14].

Study of Ommati, Farshad [15] demonstrated that AGM could alleviate hepatic injury in a rat model of obstructive jaundice via its antioxidant properties and its effects on mitochondrial indices [15]. Similarly, the antioxidant and anti-inflammatory potential of AGM has been demonstrated against hepatotoxicity induced by acetaminophen [16], D-galactosamine and lipopolysaccharide [17], and chlorpromazine [18]. Additionally, AGM may safeguard the gastrointestinal tract from the adverse effects of nonsteroidal anti-inflammatory drugs by reducing inflammation and oxidative damage [19]. AGM also counteracted the neurotoxicity associated with antipsychotic medications by diminishing neuroinflammation and oxidative stress, thereby potentially improving the safety profiles of these drugs [20]. Overall, the multifaceted protective effects of AGM highlight its potential as a therapeutic adjunct in managing drug-induced toxicity via restoring oxidative balance and inhibiting inflammation.

Collectively, we hypothesized that AGM could be a promising tool to prevent TAC-induced testicular toxicity in rats.

## 2. Materials and Methods

### 2.1. Animals

Thirty-six male Wistar rats, weighing 200–250 g (approximately 7–8 weeks old), were obtained from the “Medical Experimental Research Center”, Faculty of Medicine, Mansoura University, Egypt. The rats were placed in plastic cages at temperature of ~25 °C and allowed free access to food and water and were sustained on a 12 h light/dark cycle. Animal care and procedures were carried out in accordance with the National Institutes of Health (NIH) guidelines and approved by the “Animal Care and Use Committee”, Mansoura University, Egypt, as a part from research project with the code number MU-ACUC (PHARM.PhD.23.02.16).

### 2.2. Chemicals and Drugs

TAC was obtained as a Prograf^®^ capsule from a local pharmaceutical company (Astellas Pharma Inc., Chuo-Ku, Japan) and was suspended in carboxymethyl cellulose (CMC) for intraperitoneal injection. Agmatine^®^ powder was obtained from Nutricost (Vineyard, UT, USA) and was dissolved in distilled water for oral administration.

### 2.3. Experimental Design

Rats were randomly divided into 6 groups (6 rats each) as illustrated in Table 1:

The dose of TAC was selected based on previous studies [21,22,23] and the pilot study using three different regimens of TAC: (1) 5 mg/kg, oral; (2) 7 mg/kg, oral; (3) 5 mg/kg, i.p.; and the study indicated that 5 mg/kg *i.p.* is the most effective regimen to induce testicular injury.

On day 15, the rats were anaesthetized with thiopental sodium (40 mg/kg, i.p.). Blood samples were collected from the retro-orbital plexus and centrifuged at 4000 rpm for 15 min to collect serum for assessment of total testosterone (TT) and luteinizing hormone (LH). Then, the epididymes and testes were isolated, rinsed in ice-cold saline, dried, and weighed. The epididymes were collected for sperm analyses. The right testis was homogenized in phosphate-buffered saline (10% *w*/*v*) for biochemical and molecular assessment. The left testes were kept in Bouin’s fixative solution for histopathological and immunohistochemical analysis.

### 2.4. Semen Analysis

Cauda epididymis was dissected out, minced in physiological saline, and then incubated for 30 min at 37 °C to allow spermatozoa to leave the epididymal tubules for sperm analysis [24]. Sperm concentration and viability were analyzed for each sample. For sperm counting, every 0.5 mL of sperm sample was diluted with 9.5 mL of fixative (50 g of sodium bicarbonate and 10 mL of 35% (*v*/*v*) formalin (1:20)); then, the hemocytometer was loaded with diluted sample, allowing the spermatozoa to settle in a humid chamber, and then the spermatozoa were counted in the middle square using a 200x lens. The sperm count was presented as the concentration of million/mL. Sperm viability was calculated as a percent using the one step eosin-nigrosine procedure [25].

### 2.5. Assessment of Serum TT and LH Levels

Serum levels of TT (MyBioSource, San Diego, CA, USA, MBS282195) and LH (MyBioSource, San Diego, CA, USA, MBS590031) were determined according to the instructions of the manufacturer.

### 2.6. Assessment of Oxidative Stress Status

The testicular homogenate was used to estimate MDA content (MD 25 29) as well as GSH concentration (GR 25 11) using biodiagnostic assay kits (Giza, Egypt) according to the manufacturer’s instructions.

### 2.7. Assessment of Bcl-2, Inerleukin-17 (IL-17), Hemeoxygenase-1 (HO-1), Sirtuin1 (SIRT1), and iNOS Expressions

Levels of Bcl-2 (Cusabio, Houston, TX, USA, CSB-E08854r), IL-17 (Novus Biologicals, Centennial, CO, USA, CSB-E07451r), HO-1 (Cusabio, Houston, TX, USA, CSB-E08267r), SIRT1 (BT LAB, Beijing, China, E1145Ra), and iNOS (Assay Genie, Duplin, Ireland, RTFI00088) were measured in testicular homogenate as instructed by the manufacturer.

### 2.8. Histopathological Analyses

The left testicle was fixed in Bouin’s solution for histopathological analyses. After 24 h, all samples were dehydrated in ascending concentration of alcohol (70–100%). All tissues were then cleared in xylene, embedded in paraffin, and cut by the microtome at a thickness of 3 μm and stained with hematoxylin and eosin (H&E) for the evaluation of tissue injury. Histopathological analyses were carried out by the pathologist in a blinded manner.

### 2.9. Testicular Morphometry

Morphometric analysis of micrographs of the testes was performed as previously described [25]. About 15 transverse sections of seminiferous tubules were chosen randomly and measured for each animal. The seminiferous tubular diameter and height of the seminiferous tubule epithelium were measured at 100 × magnification using the software image J version 1.51r (NIH, Bethesda, MD, USA).

### 2.10. Immunohistochemical Analyses

Immunohistochemical analyses were used for the detection of caspase-3, nuclear factor kappa B (NF-κB), and nuclear factor erythroid 2-related factor2 (Nrf2) expression in testicular sections. Antigen retrieval was performed after deparaffinization and rehydration. Tissue sections were incubated overnight at 4 °C with rat polyclonal antibodies against caspase-3 (Servicebio, Wuhan, China), NF-κB (ABclonal, Woburn, MA, USA), and Nrf2 (Servicebio, China). After incubation, the sections were washed and treated with a goat anti-rat secondary antibody (Genemed Biotechnologies, Torrance, CA, USA) for 2 h at room temperature. Diaminobenzidine (DAB) staining was used for visualization, and a light microscope (Leica, Wetzlar, Germany) was used for the examination of stained sections. To minimize background staining and confirm specificity, appropriate control experiments were conducted [26]. Immunohistochemical evaluation was performed by the pathologist in a blinded manner. ImageJ software version 1.51r (NIH, USA) was used for semiquantification of positively stained regions in a blinded manner, and the mean of six readings from the left testicular sections was calculated for each rat.

### 2.11. Statistics

Data are presented as the mean ± standard deviation (SD). The Shapiro–Wilk test was used to test normal distribution of data. One-way analysis of variance (ANOVA) followed by Tukey–Kramer’s multiple comparison post hoc test was used to measure differences among groups. The significance level was set at *p* < 0.05. Statistical analyses and graphing were carried out using Graph Pad Prism software (V 8.4.2, Graph Pad Software Inc., La Jolla, CA, USA).

## 3. Results

There were no significant differences among the control, AGM_10_, and AGM_40_ group in all measured parameters. 

### 3.1. Impact of AGM_10_ and AGM_40_ on TAC-Induced Changes in Sperm Count, Sperm Viability, Serum TT, and LH Concentration

TAC administration exhibited a significant reduction in sperm viability after 1, 2, and 3 h by 45.4%, 50.0%, and 43.0%, respectively, relative to the control group (*p* < 0.05, Table 2). Administration of AGM_10_ along with TAC did not significantly affect sperm viability after 1, 2, and 3 h (*p* > 0.05, Table 1), whereas rats treated with AGM_40_ along with TAC showed a significant increase in sperm viability after 1, 2, and 3h (*p* < 0.05, Table 2) in comparison to the TAC group. 

**Figure 1:**
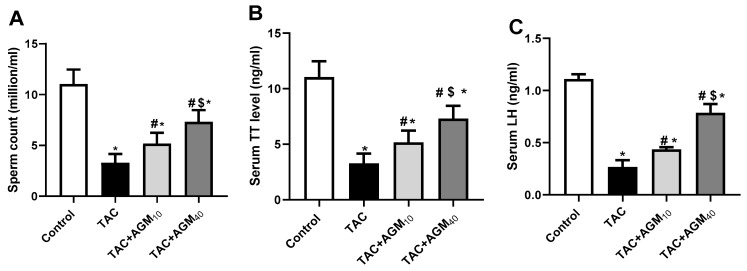
Impact of AGM_10_ and AGM_40_ on TAC-induced changes in sperm count, serum TT, and LH concentration. TAC was administered along with AGM for 14 days. (**A**) Sperm count, (**B**) serum TT, and (**C**) LH concentration. Data are expressed as means ± SD (n = 6 rats/group). Mean values were compared using one-way ANOVA followed by post hoc Tukey’s multiple comparison test. * indicates significance at *p* value of <0.05 vs. control group; ^#^ indicates significance at *p* value of <0.05 vs. TAC group; and ^$^ indicates significance at *p* value of <0.05 vs. TAC + AGM_10_ group. AGM: agmatine; CTR: control; LH: luteinizing hormone; TAC: tacrolimus; TT: total testosterone.

Administration of TAC for 14 days exhibited a significant decrease in sperm count by 70.3% compared to the control group (*p* < 0.05, Figure 1A). Rats treated with AGM_10_ or AGM_40_ along with TAC significantly increased sperm count 1.6- and 2.2-fold, respectively, relative to the TAC group (*p* < 0.05, Figure 1A). The elevation of sperm count was more significant in the TAC + AGM_40_ group than the TAC + AGM_10_ group (*p* < 0.05, Figure 1A).

TAC administration for 14 days significantly decreased serum TT level by 67.5% compared to the control group (*p* < 0.05, Figure 1B). Rats treated with AGM_10_ or AGM _40_ along with TAC significantly increased serum TT level 1.2- and 1.8-fold, respectively, in comparison to the TAC group (*p* < 0.05, Figure 1B). The elevation of serum TT level was more significant in the TAC + AGM_40_ group than the TAC + AGM_10_ group (*p* < 0.05, Figure 1B).

TAC administration exhibited a significant reduction in LH levels by 76.4% relative to the control group (*p* < 0.05, Figure 1C). Rats treated with AGM_10_ or AGM_40_ significantly increased LH levels 1.9- and 2.9-fold, respectively, compared to the TAC group (*p* < 0.05, Figure 1C). However, the effect of TAC + AGM_40_ on LH levels was more pronounced than in TAC + AGM_10_ (*p* < 0.05, Figure 1C).

### 3.2. Impact of AGM_10_ and AGM_40_ on TAC-Induced Histopathological Changes

**Figure 2:**
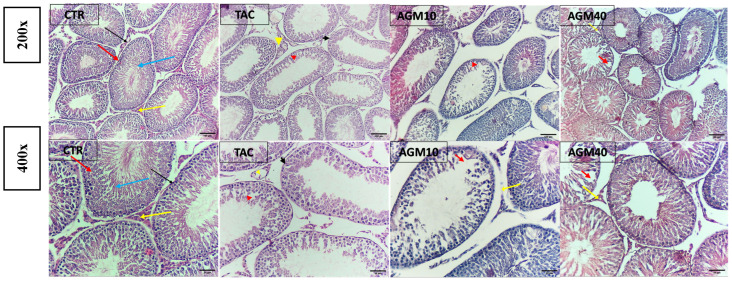
Impact of AGM_10_ and AGM_40_ on TAC-induced histopathological changes. Microscopic images of H&E-stained testicular sections. Upper panel: magnification 100x (scale bar 100 µm), and lower panel: magnification 200× (scale bar 50 µm). Black arrow: spermatogonia; red arrow: spermatocytes; blue arrow: rounded and longitudinal spermatids; yellow arrow: Leydig cell; black arrowhead: irregularly shaped seminiferous tubule; red arrowhead: sloughing of germinal epithelium; and yellow arrowhead: vacuolation with pyknotic or necrosis of Leydig cell. AGM: agmatine; CTR: control; TAC: tacrolimus.

H&E micrographs of testicular sections showed normal features of the testis with an almost rounded seminiferous tubule lined by several layers of spermatogenic cells, and a lumen filled with sperm in the control group. On the other hand, TAC administration resulted in the loss of the seminiferous tubule’s normal shape, sloughing of the germinal epithelium, vacuolation with the pyknotic Leydig cell, and a decrease in the number of spermatocytes. Testicular sections from rats treated with AGM_10_ along with TAC showed a partial restoration of the seminiferous tubule’s normal shape, mild sloughing of the germinal epithelium, and necrosis of the Leydig cell, whereas testicular sections from rats treated with AGM_40_ along with TAC exhibited restoration of the seminiferous tubule’s normal shape, minimal sloughing of the germinal epithelium, and minimal vacuolation with the pyknotic Leydig cell (Figure 2).

### 3.3. Impact of AGM10 and AGM40 on TAC-Induced Changes in Morphometry of the Seminiferous Tubules

The height of the seminiferous epithelium was 70.6 ± 12.3 µm in the control group (Table 3). TAC administration significantly decreased height of the seminiferous epithelium compared to the height of the control group (*p* < 0.05). Rats treated with AGM_10_ or AGM_40_ along with TAC significantly increased the height of the seminiferous epithelium (*p* < 0.05). 

The average diameter of the seminiferous tubules was 244.4± 16.8 µm in the control group (Table 3). TAC administration significantly decreased the diameter of the seminiferous tubules compared to the diameter in the control group (*p* < 0.05). Rats treated with AGM_10_ or AGM_40_ along with TAC significantly increased the diameter of the seminiferous tubules (*p* < 0.05).

### 3.4. Impact of AGM_10_ and AGM_40_ on TAC-Induced Changes in Oxidative Stress Status

**Figure 3:**
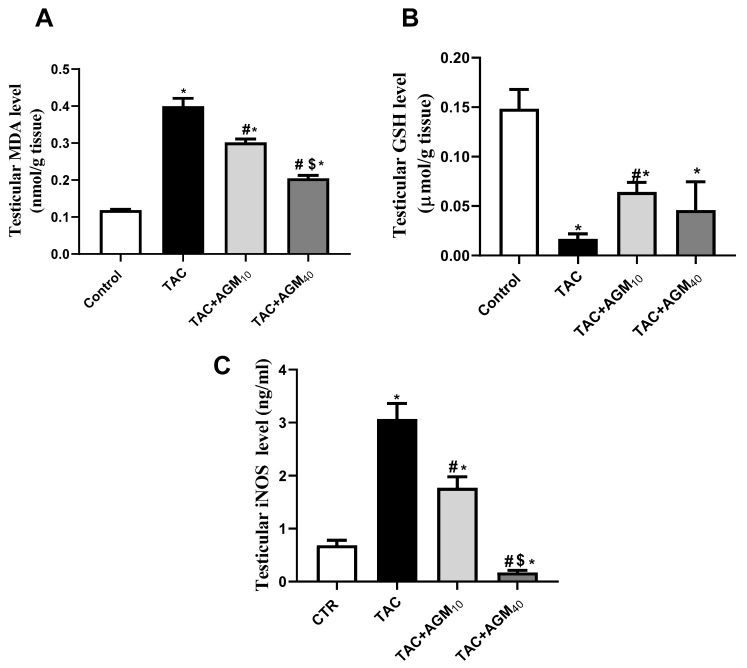
Impact of AGM_10_ and AGM_40_ on TAC-induced changes in oxidative status. (**A**) MDA content, (**B**) GSH level, and (**C**) iNOS level. TAC was administered along with AGM_10_ or AGM_40_ for 14 days. Data are expressed as means ± SD (n = 6 rats per group). Mean values were compared via one-way ANOVA followed by post hoc Tukey’s multiple comparison test. * indicates significance at *p* value of <0.05 vs. CTR group. ^#^ indicates significance at *p* value of <0.05 vs. TAC group and ^$^ indicates significance at *p* value of <0.05 vs. TAC + AGM_10_ group. AGM: agmatine; CTR: control; GSH: reduced glutathione; iNOS: inducible nitric oxide; MDA: malondialdehyde; synthase; TAC: tacrolimus.

Administration of TAC for 14 days exhibited a 3.4-fold increase in MDA content relative to control group (*p* < 0.05, Figure 3A). Treatment with AGM_10_ and AGM_40_ along with TAC resulted in a significant reduction of MDA content by 24.3% and 48.7%, respectively, as compared to the TAC group (*p* < 0.05, Figure 3A). The effect was more significant in the TAC + AGM_40_ group than the TAC + AGM_10_ group (*p* < 0.05, Figure 3A).

TAC also significantly reduced GSH levels by 88.7% as compared to the control group (*p* < 0.05, Figure 3B). Rats treated with AGM_10_ and AGM_40_ along with TAC exhibited a significant increase in GSH levels by 3.8- and 2.7-fold, respectively, as compared to the TAC group (*p* < 0.05, Figure 3B).

Administration of TAC significantly increased the iNOS level 4.5-fold in comparison to the control group (*p* < 0.05, Figure 3C). Rats treated with AGM_10_ and AGM_40_ with TAC for 14 days significantly decreased iNOS levels by 42.3% and 94.3%, respectively, as compared to the TAC group (*p* < 0.05, Figure 3C). The impact was more significant in the TAC + AGM_40_ group than the TAC + AGM_10_ group (Figure 3C).

### 3.5. Impact of AGM_10_ and AGM_40_ on TAC-Induced Changes in Nrf2, HO-1, and SIRT1 Expression

**Figure 4:**
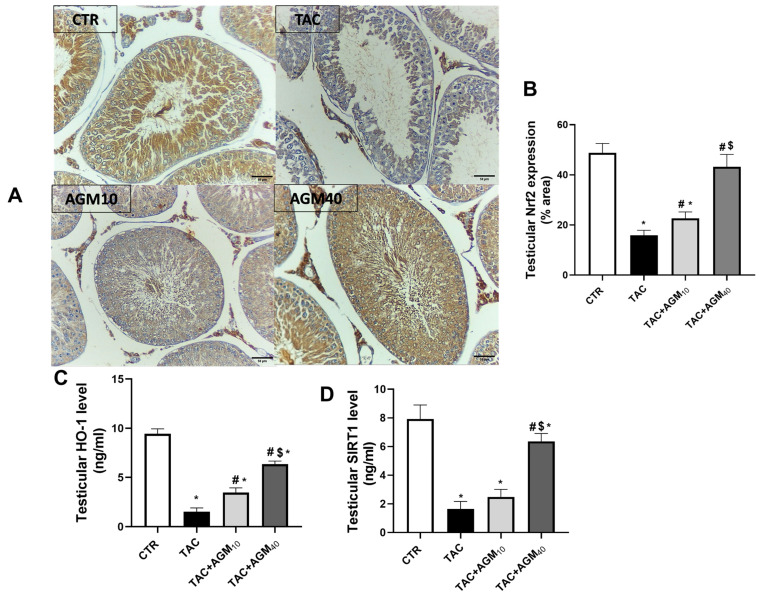
Impact of AGM_10_ and AGM_40_ on TAC-induced changes in Nrf2 expression and HO-1 and SIRT1 levels. TAC was administered along with AGM_10_ or AGM_40_ for 14 days. (**A**) Photomicrograph of immunostained testicular sections against Nrf2 (magnification 200× and scale bar 50 µm), (**B**) semiquantification of Nrf2, (**C**) HO-1 level, and (**D**) SIRT1 level. Data are expressed as means ± SD (n = 6 rats per group). Mean values were compared via one-way ANOVA followed by post hoc Tukey’s multiple comparison test. * indicates significance at *p* value of <0.05 vs. CTR group, ^#^ indicates significance at *p* value of <0.05 vs. TAC group, and ^$^ significance at *p* value of <0.05 vs. TAC + AGM_10_ group. AGM: agmatine; CTR: control; HO-1: Heme Oxygenase-1; Nrf2: nuclear factor erythroid 2; SIRT1: Nad-dependent protein deacetylase sirtuin 1; TAC: tacrolimus.

Immunostaining of testicular sections against Nrf2 showed high immunoreactivity in spermatocytes, spermatogonia, spermatids, Sertoli cells, and Leydig cells in the control group (Figure 4A). However, the TAC group showed low immunoreactivity in the spermatocytes, spermatogonia, spermatids, and Leydig cells (Figure 4A). The TAC + AGM_10_ group displayed mild immunoreactivity in the spermatocytes and spermatids (Figure 4A). The TAC + AGM_40_ group showed moderate immunoreactivity in the spermatocytes, spermatogonia, and spermatids (Figure 4A).

Semiquantitative analysis showed that administration of TAC for 14 days significantly decreased Nrf2 expression by 76.3% compared to the control group (*p* < 0.05, Figure 4B). However, administration of AGM_10_ and AGM_40_ along with TAC significantly increased Nrf2 expression 1.4- and 1.6-fold, respectively, compared to the TAC group (*p* < 0.05, Figure 4B).

TAC administration also resulted in a significant decrease in HO-1 levels by 84% as compared to the control group (*p* < 0.05, Figure 4C). Administration of AGM_10_ or AGM_40_ along with TAC significantly increased HO-1 levels 2.3-fold and 4.2-fold, respectively, as compared to the TAC group (*p* < 0.05, Figure 4C). The impact was more significant in the TAC + AGM_40_ group than the TAC + AGM_10_ group (*p* < 0.05, Figure 4C).

TAC significantly decreased SIRT1 levels by 79.1% as compared to the control group (*p* < 0.05, Figure 4D). Rats treated with AGM_10_ along with TAC did not significantly increase SIRT1 levels compared to the TAC group (*p* < 0.05, Figure 4D). Administration of AGM_40_ along with TAC significantly increased SIRT1 levels 3.9-fold as compared to the TAC group (*p* < 0.05, Figure 4D). The impact was more significant in the TAC + AGM_40_ group than the TAC + AGM_10_ group (*p* < 0.05, Figure 4D)

### 3.6. Impact of AGM_10_ and AGM_40_ on TAC-Induced Changes in NF-κB and IL-17 Expressions

**Figure 5:**
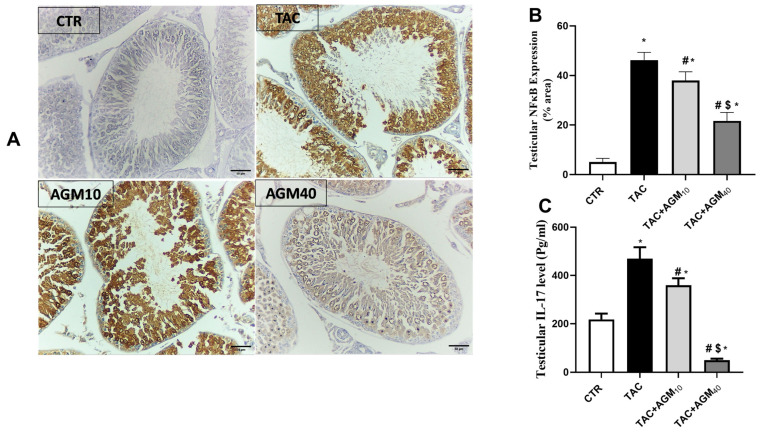
Impact of AGM_10_ and AGM_40_ on TAC-induced changes in NF-κB and IL-17 levels. TAC was administered along with AGM_10_ or AGM_40_ for 14 days. Testes were collected to assess the following: (**A**) photomicrograph of immunostained testicular sections against NF-κB (magnification 200× and scale bar 50 µm), (**B**) NF-κB expression, and (**C**) IL-17 levels. Data are expressed as means ± SD (n = 6 rats/group). Mean values were compared via one-way ANOVA followed by post hoc Tukey’s multiple comparison test. * indicates significance at *p* value of <0.05 vs. CTR group. ^#^ indicates significance at *p* value of <0.05 vs. TAC group, and ^$^ indicates significance at *p* value of <0.05 vs. TAC + AGM_10_ group. AGM: agmatine; CTR: control; IL-17: interleukin 17; NF-κB: nuclear factor kappa B; TAC: tacrolimus.

Immunostaining of testicular sections against NF-κB showed mild immunoreactivity in spermatocytes, spermatogonia, or spermatids in the control group (Figure 5A). However, the TAC group showed high immunoreactivity in the spermatocytes, spermatogonia, spermatids, and Leydig cells (Figure 5A). Yet, the TAC + AGM_10_ group showed moderate immunoreactivity in the spermatocytes, spermatogonia, and spermatids (Figure 5A). The TAC + AGM_40_ group displayed mild immunoreactivity in the spermatocytes, spermatogonia, and spermatids (Figure 5A).

Semiquantitative analysis for NF-κB showed that TAC administration significantly increased NF-κB expression 9.1-fold compared to the control group (*p* < 0.05, Figure 5B). Yet, administration of AGM_10_ and AGM_40_ along with TAC significantly decreased NF-κB expression by 17.9% and 53%, respectively, compared to the TAC group (*p* < 0.05, Figure 5B). The effect was more so in the TAC + AGM_40_ group than the TAC + AGM_10_ group (*p* < 0.05, Figure 5B).

TAC also significantly increased IL-17 levels 2.1-fold compared to the control group (*p* < 0.05, Figure 5C). Yet, rats treated with AGM_10_ and AGM_40_ along with TAC exhibited significantly decreased IL-17 levels by 23.5% and 89.0%, respectively, as compared to the TAC group (*p* < 0.05, Figure 5C). The impact was more significant in the TAC + AGM_40_ group than the TAC + AGM_10_ group (*p* < 0.05, Figure 5C).

### 3.7. Impact of AGM10 and AGM40 on TAC-Induced Changes in Caspase-3 and Bcl-2 Expression

**Figure 6:**
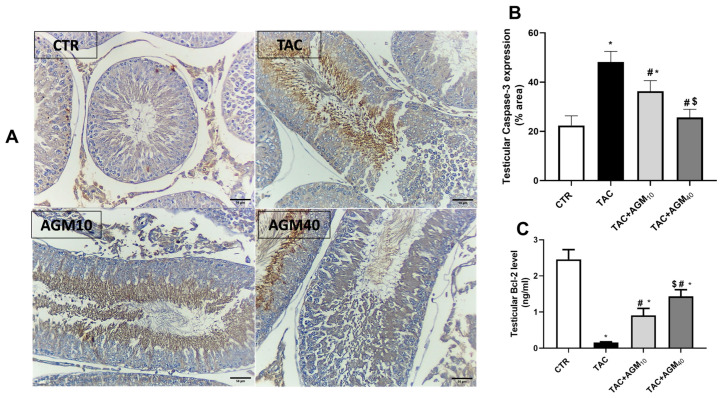
Impact of AGM_10_ and AGM_40_ on TAC-induced changes in caspase 3 and Bcl-2 expression. TAC was administered along with AGM_10_ or AGM_40_ for 14 days. (**A**) Photomicrograph of immunostained testicular sections against caspase-3 (magnification 200× and scale bar 50 µm), (**B**) Caspase-3 expression, and (**C**) Bcl-2 levels. Data are expressed as means ± SD (n = 6 rats/group). Mean values were compared via one-way ANOVA followed by post hoc Tukey’s multiple comparison test. * indicates significance at *p* value of <0.05 vs. CTR group. ^#^ indicates significance at *p* value of <0.05 vs. TAC group, and ^$^ indicates significance at *p* value of <0.05 vs. TAC + AGM_10_ group. AGM: agmatine; CTR: control; TAC: tacrolimus.

Immunostaining of testicular sections against caspase-3 showed minimal immunoreactivity in spermatocytes and spermatogonia of the control group (Figure 6A). However, the TAC group showed high immunoreactivity in the spermatocytes, spermatogonia, and spermatids (Figure 6A). Yet, the TAC + AGM_10_ group revealed moderate immunoreactivity in the spermatocytes, spermatogonia, and spermatids (Figure 6A). The TAC + AGM_40_ group revealed mild immunoreactivity in the spermatocytes and spermatids (Figure 6A).

Semiquantitative analysis for caspase-3 showed that TAC administration significantly increased caspase-3 expression 2.1-fold as compared to the control group (*p* < 0.05, Figure 6B). Nonetheless, administration of AGM_10_ and AGM_40_ along with TAC resulted in a significant decrease in caspase-3 expression by 24.5% and 64.8%, respectively, as compared to the TAC group (*p* < 0.05, Figure 6B). The effect was more so in the TAC + AGM_40_ group than the TAC + AGM_10_ group (*p* < 0.05, Figure 6B).

Administration of TAC for 14 days significantly decreased Bcl-2 levels by 93.6 % as compared to the control group (*p* < 0.05, Figure 6C). Yet, administration of AGM_10_ and AGM_40_ along with TAC significantly increased Bcl-2 levels 5.8- and 9.1-fold, respectively as compared to the control group (*p* < 0.05, Figure 6C). The impact was more significant in TAC + AGM_40_ group than the TAC + AGM_10_ group (*p* < 0.05, Figure 6C).

## 4. Discussion

Results of the current study demonstrated that AGM, a potent inhibitor of iNOS, could ameliorate TAC-induced testicular injury by restoring the oxidant/antioxidant balance, and this effect was associated with modulating the Nrf2/NF-κB/SIRT1/HO-1 pathway, resulting in a reduction in oxidative damage, inflammation, apoptosis, and autophagy by regulating IL-17, caspase-3, and Bcl-2 expression.

TAC treatment in this study resulted in a significant drop in serum testosterone, sperm viability, and sperm count, suggesting the impairment of the reproductive system. TAC had a significant impact on the hypothalamic–pituitary–gonadal axis, as demonstrated by a decline in the level of serum LH, causing spermatogenesis impairment. TAC-induced testicular injury was further confirmed by histopathology, showing sloughing of the germinal epithelium, a loss of the seminiferous tubule’s normal shape, vacuolation, and Leydig cells with pyknotic nuclei. The harmful effects of TAC on the male reproductive system have been investigated by few studies, and the findings include a decrease in testicular weight, a decrease in epithelial thickness in the seminiferous tubules [26,27], a drop in the number of spermatids, and an increase in the number of malformed spermatozoa [28], consistent with our findings.

Both low and high doses of AGM showed a dose-dependent increase in sperm viability, sperm count, LH concentrations, and testosterone levels, suggesting a possible protective role of AGM against TAC-induced testicular damage that was further assured by histopathological analysis.

Testicular injury by TAC is concomitant with a multifactional etiology; however, oxidative stress is regarded as the most common mechanism of TAC-induced testicular injury. Apoptotic cell death is closely related to TAC-induced oxidative stress [29]. A correlation between ROS and male infertility has been documented in some reports [30]. Consequently, several studies have reported that antioxidants could improve sperm parameters [31,32,33].

Studies have demonstrated that TAC administration caused a significant increase in MDA levels, an indicator of lipid peroxidation and a sign of oxidative stress in liver and kidney tissues [34,35,36]. Moreover, TAC also increased iNOS activity, leading to elevated production of nitric oxide and peroxynitrite [37]. GSH is an effective antioxidant present in cells that plays a role in protecting against oxidative stress and is involved in multiple metabolic processes [35] and whose expression has been proven to decrease post treatment with TAC [35,36]. In this study, the administration of TAC for 14 days resulted in a crucial elevation in MDA and iNOS content compared to the control group and a significant decrease in GSH, suggesting a marked oxidant/antioxidant imbalance. On the other hand, groups treated with AGM_10_ or AGM_40_ along with TAC exhibited a substantial decrease in MDA and iNOS and a significant increase in GSH levels in testicular tissues as compared to the TAC group, suggesting the antioxidant potential of AGM. These findings are consistent with previous studies that had supported that AGM shows clear therapeutic potential in numerous pathological circumstances by inhibiting oxidative stress and inflammation [38,39]. Thus, this would result in precluding the onset of oxidative stress.

SIRT1, a longevity gene, plays a vital role in regulating various biological processes, including cellular senescence, as well as regulating oxidative stress, inflammation, mitochondrial function, immune responses, cell apoptosis, and cellular differentiation and proliferation [35,40,41]. SIRT1 is a protein deacetylase that controls many survival activities by deacetylating histones and a number of transcription factors, including eNOS and Nrf2, which are involved in apoptosis and the generation of ROS [42]. Also, Vomund et al., 2017 proved that downregulation of SIRT1 under oxidative stress most likely leads to less deacetylation of Nrf2 [43]. SIRT1 enhances the activation of Nrf2, as well as its downstream key antioxidant gene HO-1, to protect cells against oxidative damage by deacetylating the transcription coactivator PPARγ coactivator-1α [35]. Nrf2 is the crucial regulator that counteracts ROS generation by activating antioxidant cascades [35]. Downstream of SIRT1, deacetylated PGC-1α in turn facilitates the upregulation of Nrf2 and antioxidant-related genes [35]. Normally, Keap1, the repressor of Nrf2, binds to Nrf2 in a complex, sequestering it in the cytoplasm [35,36]. Upon stimulation by oxidative and electrophilic chemical signals, Nrf2 is released from Keap1 and translocates into the nucleus, where it binds to antioxidant responsive elements (AREs) [35,36]. Many cellular protective genes, such as HO-1 and NAD(P)H quinine oxidoreductase-1(NQO1) [35,36], are target genes of nuclear Nrf2. Experimental evidence has shown that HO-1 plays an important role in host defense against ROS generation and oxidative injury [35,36]. It also contributes to the anti-inflammatory activity of cells and tissues [35]. Evidence indicates that the decreased expression of HO-1 and increased levels of thiobarbituric acid reactive substances observed in T2DM could be a consequence of a reduced expression of Nrf2 [44]. Lamiri et al., 2021 showed that linear regression analysis revealed a strong and positive correlation between the changes in the Nrf2 and HO-1 expression levels [44].

The SIRT1 and NF-κB signaling are evolutionarily conserved mechanisms in the maintenance of cellular homeostasis, and their interaction regulates the inflammatory response [45]. The nature of this relationship is antagonistic, so that SIRT1 is capable of inhibiting NF-κB signaling and vice versa [46]. Evidence reports that TAC-induced oxidative damage with the subsequent release of damage-associated molecular patterns in the mitochondrial and nuclear DNA provokes inflammatory cells, leading to stimulation of the transcription factor NF-κB and the generation and release of inflammatory mediators including IL-17. TAC is also reported to decrease the expression of Nrf2/HO-1, an essential pathway for shielding cells from inflammation and oxidative stress [47]. Also, previous studies have shown the important involvement of NF-κB in the inflammation, apoptosis, and oxidative damage induced by TAC. When TAC decreases Nrf2 and HO-1 expression, it leads to increased oxidative stress [48] and spermatocyte injury [49]. In addition, the absence of SIRT1 has been reported to contribute to failure of the HPG axis leading to reduced LH, therefore impeding the production of testosterone and estrogen. The reduction in sex hormones leads to compromised sexual behavior and aberrant spermatogenesis [50].

Therefore, we evaluated whether oxidant/antioxidant imbalance is related to imbalance in the SIRT1/Nrf2/NF-κB/Bcl-2 pathway. In this context, TAC administration in the present study markedly decreased SIRT1 and Nrf2 and increased NF-κB expression, which could explain, in part, the induction of oxidative stress. Also, NF-κB is considered as a main regulator of the inflammatory response owing to its ability to regulate the transcription of genes involved in the establishment of immune and inflammatory responses including IL-17 [51,52]. The overproduction of IL-17 leads to chronic inflammation and tissue damage [53,54]. IL-17 was reported to affect the blood–testicular barrier and thus might trigger an inflammatory cascade in the testis. IL17 could disrupt the Sertoli cell barrier. IL17 could enhance the recruitment of other immune cells, specifically macrophages, to the testes, and IL17 itself leads to harmful effect on germ cells and the seminiferous epithelium [44]. Numerous investigations have demonstrated that TAC markedly upregulated the expression of proinflammatory IL-10 and IL-17 while downregulating the expression of anti-inflammatory IL-4 and IFN-γ [55,56]. The findings of the current study revealed a substantial increase in the expression of IL-17 in the TAC-treated group.

Also, SIRT1 has a fundamental role in the regulation of apoptosis. It functions as a NAD+-dependent deacetylase, modulating the activity of various apoptotic factors. By deacetylating and activating key proteins such as p53, SIRT1 can suppress apoptosis in response to stress. It also promotes cell survival via enhancing the expression of anti-apoptotic proteins like Bcl-2 and inhibiting pro-apoptotic factors such as Bax [57,58]. The SIRT1-deficient testes revealed increased apoptosis in male germ cells and other testicular cells [59]. Male germ cell death in SIRT1^−/−^ mice was related to higher p53 activity, as testicular apoptosis is dependent on acetylation-mediated p53 activity [50]. Overall, SIRT1 serves as a critical regulator of apoptosis, promoting cell resilience and longevity under stress conditions.

A previous study revealed that TAC greatly decreased the expression of antiapoptotic factors like Bcl-2 in spermatogenic cells, and significantly changed the expression of proteins linked to apoptosis, such as caspase-3, as compared to the control group [60]. Consistently, our study demonstrated an increase in caspase-3 expression in testicular sections in spermatocytes, spermatogonia, or spermatids in the TAC group along with the reduction in Bcl-2 expression.

The current study demonstrated that rats treated with AGM_10_ or AGM_40_ exhibited a significant upregulation of Nrf2, HO-1, SIRT1, and Bcl-2 expression and downregulation of NF-κB, IL-17, and caspase-3 in comparison to the TAC group. The increase in expression of HO-1 by AGM, as a potent iNOS inhibitor, could be a consequence of the increase in the expression of Nrf2 and/or decreased ROS levels as evidenced by lower MDA and iNOS and higher GSH levels. The inhibition of inflammation/oxidative stress observed with AGM through the modulation of Nrf2/HO-1 and NF-κB signaling could be a consequence of the upregulation of SIRT1. Chai et al., 2016 hypothesized that AGM inhibits the induction of ROS through AGM-mediated Nrf2/HO-1 expression [61]. Also, Freitas et al. 2015 proved that AGM protects against corticosterone effects in a hippocampal neuronal cell line by a mechanism that implicates Nrf2/HO-1 signaling [62]. These findings suggested that the antioxidant, anti-inflammatory, and antiapoptotic potential of AGM that are likely related to the inhibition of NF-κB expression and upregulation of SIRT1 and Nrf2 expression.

The limitation of this study is the focus on the acute phase of TAC-induced testicular toxicity, where longer time frame to assess the impact of AGM on chronic testicular damage needs to be considered. In addition, further validation and insight into the molecular mechanisms of AGM in the TAC-treated model is essential for future studies. Genetic approaches, such as using transgenic mice or gene knockdown/knockout techniques, should be employed to strengthen the findings and support the proposed mechanisms.

## 5. Conclusions

**Figure 7:**
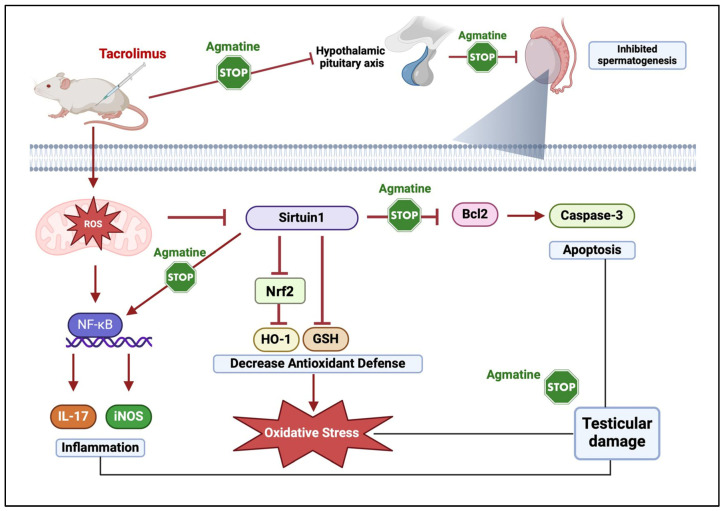
The proposed protective mechanism of AGM on TAC-induced testicular toxicity. GSH: reduced glutathione; iNOS: inducible nitric oxide synthase; IL-17: interleukin 17; HO-1: heme oxygenase-1; NF-κB: nuclear factor kappa B; Nrf2: nuclear factor erythroid 2.

In conclusion, AGM effectively attenuated testicular injuries induced by TAC as evidenced by an elevation in sperm viability, sperm count, LH concentrations, and testosterone levels that was further assured by histopathological and morphometric analyses. This protective effect of AGM might be attributed to the modulation of the Nrf2/NF-κB/SIRT1/HO-1 pathway, resulting in reducing oxidative damage, inflammation, apoptosis, and autophagy by regulating IL-17, caspase-3, and Bcl-2 expression (Figure 7).

## Figures and Tables

**Table 1:** The experimental design.

1	Control group	Rats received vehicle only for 14 days
2	AGM_10_ group	Rats received AGM (10 mg/kg/day, oral) for 14 days [13]
3	AGM_40_ group	Rats received AGM (40 mg/kg/day, oral) for 14 days [13]
4	TAC group	Rats received TAC (5 mg/kg/day, i.p.) for 14 days
5	TAC + AGM_10_ group	Rats received TAC (5 mg/kg/day, i.p.) and AGM (10 mg/kg/day, oral) for 14 days simultaneously [13]
6	TAC + AGM_40_ group	received TAC (5 mg/kg/day, i.p.) and AGM (40 mg/kg/day, oral) for 14 days simultaneously [13]

AGM: Agmatine; TAC: Tacrolimus.

**Table 2:** Impact of AGM_10_ and AGM_40_ on TAC-induced changes in sperm viability.

	Sperm Viability %		
	After 1 h	After 2 h	After 3 h
Control	52.50 ± 2.50	47.50 ± 2.50	42.50 ± 2.50
TAC	28.75 ± 4.15 *	23.75 ± 4.15 *	24.25 ± 2.44 *
TAC + AGM_10_	34.29 ± 4.32	29.00 ± 4.18	29.00 ± 9.62
TAC + AGM_40_	44.50 ± 3.71 ^#^	39.00 ± 4.18 ^#^	34.00 ± 4.18 ^#^

TAC was administered along with AGM for 14 days. Data were expressed as means ± SD (n = 6 rats/group). Mean values were compared using one-way ANOVA followed by post hoc Tukey’s multiple comparison test. * Significance at *p* value of <0.05 vs. control group; ^#^ significance at *p* value of <0.05 vs. TAC group. AGM: agmatine; CTR: control; TAC: tacrolimus.

**Table 3:** Impact of AGM_10_ and AGM_40_ on TAC-induced changes the morphometry of the seminiferous tubules.

	The Height of the Seminiferous Epithelium (µm)	The Diameter of the Seminiferous Tubules (µm)
Control	70.6 ± 12.3	244.4 ± 16.8
TAC	42.0 ± 2.0 *	182.2 ± 6.8 *
TAC + AGM_10_	59.4 ± 6.7 ^#^	198.4 ± 8.1
TAC + AGM_40_	65.4 ± 4.6 ^#^	212.6 ± 3.6 ^#^

TAC was administered along with AGM for 14 days. Data are expressed as means ± SD (n = 6 rats/group). Mean values were compared using one-way ANOVA followed by post hoc Tukey’s multiple comparison test. * indicates significance at *p* value of <0.05 vs. control group and ^#^ indicates significance at *p* value of <0.05 vs. TAC group. AGM: agmatine; CTR: control; TAC: tacrolimus.

## Data Availability

Data are contained within the article.
